# Fc-competent multispecific PDL-1/TIGIT/LAG-3 antibodies potentiate superior anti-tumor T cell response

**DOI:** 10.1038/s41598-023-36942-3

**Published:** 2023-06-18

**Authors:** Riyao Yang, Su Huang, Cai Huang, Nathan S. Fay, Yanan Wang, Saroja Putrevu, Kimberly Wright, Mohd Saif Zaman, Wenyan Cai, Betty Huang, Bo Wang, Meredith Wright, Matthew R. Hoag, Allison Titong, Yue Liu

**Affiliations:** 1 Ab Therapeutics Inc., 3541 Investment Blvd., Suite 2, Hayward, CA 94545 USA; 2 Ab Studio Inc., 3541 Investment Blvd., Suite 3, Hayward, CA 94545 USA

**Keywords:** Cancer immunotherapy, Antibody therapy

## Abstract

The landscape of current cancer immunotherapy is dominated by antibodies targeting PD-1/PD-L1 and CTLA-4 that have transformed cancer therapy, yet their efficacy is limited by primary and acquired resistance. The blockade of additional immune checkpoints, especially TIGIT and LAG-3, has been extensively explored, but so far only a LAG-3 antibody has been approved for combination with nivolumab to treat unresectable or metastatic melanoma. Here we report the development of a PDL1 × TIGIT bi-specific antibody (bsAb) GB265, a PDL1 × LAG3 bsAb GB266, and a PDL1 × TIGIT × LAG3 tri-specific antibody (tsAb) GB266T, all with intact Fc function. In in vitro cell-based assays, these antibodies promote greater T cell expansion and tumor cell killing than benchmark antibodies and antibody combinations in an Fc-dependent manner, likely by facilitating T cell interactions (bridging) with cancer cells and monocytes, in addition to blocking immune checkpoints. In animal models, GB265 and GB266T antibodies outperformed benchmarks in tumor suppression. This study demonstrates the potential of a new generation of multispecific checkpoint inhibitors to overcome resistance to current monospecific checkpoint antibodies or their combinations for the treatment of human cancers.

## Introduction

Checkpoint inhibitors reactivate T cells by blockade checkpoint receptor/ligand interactions that restrict T cell activity^1^. Several checkpoint inhibitor antibodies targeting CTLA-4 and PD-1/PD-L1 have been approved and have shown efficacy for a variety of tumor indications^2^. Responders to such agents often exhibit durable remission, which is rarely seen for other therapies^3^. However, many cancer patients do not benefit from such treatments due to primary or acquired resistance^4,5^.

The landscape of checkpoint inhibitors is still dominated by antibodies that target PD-1 or PD-L1, although the potential of targeting other immune checkpoints are also being actively investigated^2,6,7^. Among those TIGIT and LAG-3 are two distinct immune checkpoints that contribute to T cell exhaustion^1,8,9^. TIGIT is expressed in T cells and NK cells, and inhibits T cell activation mainly by competing for CD155 binding with CD226, a T cell stimulatory receptor^8,10–13^. LAG-3 is expressed on CD4 and CD8 T cells, and suppresses T cells once it is engaged by one of its ligands including MHC-II^14,15^, FGL-1^16^, or galectin-3^17,18^. These two immune checkpoints are now emerging as promising targets in the post-PD-1/CTLA-4 era in cancer immunotherapy. Strong preclinical evidence supports blocking the TIGIT pathway for cancer immunotherapy^12,19–22^, although a recent Phase III clinical trial of anti-TIGIT tiragolumab combined with anti-PD-L1 (Tecentriq) failed. Much remains to be learned about the LAG-3 pathway, including the relative importance of its three potential ligands. However, the clinical efficacy of LAG-3 inhibition has been attested, and an anti-LAG-3 antibody (relatlimab) has been approved by the FDA in combination therapy with anti-PD-1 (nivolumab) for the management of patients with unresectable or metastatic melanoma^23^. These recent developments highlight the promise of simultaneously targeting PD-1/PD-L1 and TIGIT or LAG-3 pathways for cancer immunotherapy.

Although combination immune checkpoint therapy is currently the prevailing strategy to overcome resistance to anti-PD-1/PD-L1 therapy, new technology platforms now allow for the development of a different class of therapeutic antibodies, i.e., multispecific antibodies, which can target different epitopes on two or more checkpoint molecules^24–28^. In addition to blocking multiple inhibitory pathways, such multispecific agents can have additional MOAs, including cell–cell bridging via the crosslinking of antigens on neighboring cells to achieve greater T cell activation and/or cancer cell killing^24–27^. Another factor to consider when designing therapeutic antibodies is whether to retain Fc function, which plays important roles through ADCC, ADCP, and other mechanisms that contribute to the efficacy of these antibodies^29,30^. It has been shown that the activities of PD-L1 antibodies rely on FcγR engagement, while those of anti-PD-1 Abs are FcgR-independent^31^. In addition to inducing effector functions such as ADCC, ADCP, and CDC, Fc engagement with FcγR on APCs contribute to stronger T cell activation^32,33^. The importance of Fc binding for TIGIT antibody efficacy has been a matter of debate, but results from most recent studies are in favor of retaining or enhancing Fc binding^22,32,33^.

We have developed multispecific antibodies targeting PD-L1, TIGIT, and LAG-3 and evaluated them with carefully designed in vitro and in vivo assays, which show that these antibodies efficiently promote T cell activation and cancer cell killing and suppress tumor growth. We further show that their superior efficacy compared to antibody combinations depends on the function of the Fc region and the presence of monocytes. As monocytes/macrophages are a major component of tumor-infiltrating immune cells along with T cells in the tumor microenvironment, our antibodies are likely to outperform current benchmark antibodies in cancer immunotherapy.

## Results

### Generation of GB265, 266, and 266T multispecific antibodies and their target binding/blocking characteristics

**Figure 1:**
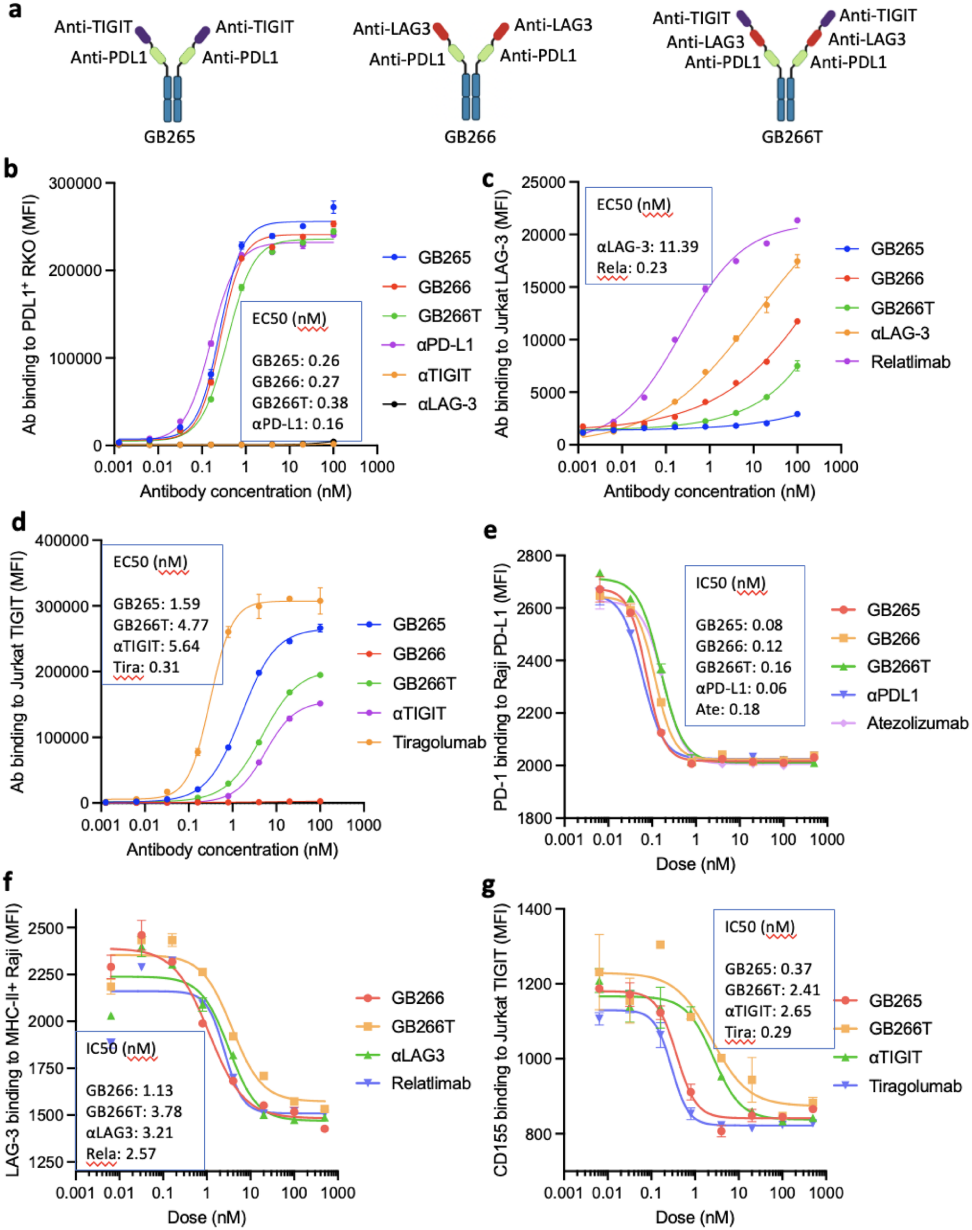
Target binding and blocking characteristics of GB265, GB266, and GB266T antibodies. (**a**) Configuration of GB265, GB266, and GB266T antibodies. (**b–d**) Target binding characteristics of GB265, GB266, and GB266T antibodies. Indicated target cells were incubated with GB265, GB266, GB266T, or control antibodies. Bound antibodies were then detected with a fluorescence-labeled secondary antibody specific for human IgG Fc by flow cytometry. The RKO colon cancer cell line expresses endogenous PD-L1. (**e–g**) Target blocking characteristics of GB265, GB266, and GB266T antibodies. Indicated target cells were incubated with GB265, GB266, GB266T, or control antibodies, in the presence of biotinylated PD-1, LAG-3, or CD155. Bound biotinylated proteins were then detected with fluorescence-labeled streptavidin by flow cytometry. αTIGIT, αLAG, and αPDL1 represent parental in-house antibodies (bivalent monospecific VHH molecules expressed with an IgG1 Fc). Assays were performed in duplicates and data are presented as mean ± SD.

We discovered llama single domain antibodies (VHHs) that bind to TIGIT or LAG-3 by screening our in-house naïve and synthetic humanized llama VHH phage libraries with recombinant TIGIT or LAG-3 protein. The lead binders were further assessed for their ability to block CD155/TIGIT and MHC-II/LAG-3 interactions, respectively. VHHs with high binding affinity and blocking ability were selected and fused into a single PDL1 × TIGIT bsAb (GB265), PDL1 × LAG3 bsAb (GB266) or a PDL1 × LAG3 × TIGIT tsAb (GB266T), with the aid of our computer-aided antibody design (CAAD) platform for optimization^34–38^ (Fig. 1a). The sequence of the PD-L1 binding domain of GB265, GB266 and GB266T originated from the Alphamab anti-PD-L1 antibody envafolimab^39^. T cells that express LAG-3 or TIGIT were generated by transfection of Jurkat cells with either protein. Binding characteristics of the three antibodies to cell-associated PD-L1, TIGIT, and LAG-3 are shown in Fig. 1b–d. Using RKO expressing endogenous PD-L1, we found that GB265, GB266, and GB266T largely retain the high PD-L1 binding activity of the parental antibody (Fig. 1b). With TIGIT-transfected Jurkat cells, we found that GB266T has a binding affinity for TIGIT similar to that of ABT’s parental anti-TIGIT antibody, while that of GB265 is notably higher, and all of them exhibited significantly lower affinity than Roche’s tiragolumab (Fig. 1c). Improved binding of GB265 to TIGIT compared to the parental TIGIT antibody suggests that the PD-L1-binding domain in GB265 exerts a positive effect on TIGIT binding. The LAG-3-binding activity of GB266T is lower than that of GB266, and both are markedly lower than that of ABT’s parental LAG-3 antibody, as well as BMS’s relatlimab (Fig. 1b–d). Overall, these results show that GB265, GB266, and GB266T antibodies have high affinities for PD-L1, similar to that of the monospecific parental antibody envafolimab, and relatively low affinities for TIGIT and LAG-3 compared to that of tiragolumab and relatlimab, respectively. As strong antibody binding to LAG-3 and TIGIT can lead to Fc-mediated ADCC/ADCP of T cells, such “imbalanced” binding characteristics (high affinities for PD-L1 and moderate to low affinities for LAG-3 and TIGIT) can be desirable for efficacy/safety balance.

Consistent with their high PD-L1 binding affinities (Fig. 1b), GB265, GB266, and GB266T efficiently blocked PD-1/PD-L1 binding, compared with the parental Alphamab anti-PD-L1 envafolimab, with IC50’s around 0.1 nM (Fig. 1e). Interestingly, despite their low LAG-3 binding affinities (Fig. 1c), GB266 and GB266T blocked LAG3/MHC-II binding with IC50’s similar to those of the parental LAG3 antibody and the BMS LAG-3 antibody relatilimab (Fig. 1f). Consistent with the binding data, GB265 and the Roche TIGIT antibody tiragolumab blocked CD155/TIGIT binding more efficiently (IC50’s ~ 0.3 nM) than GB266T and the parental TIGIT antibody (IC50’s ~ 2.5 nM) (Fig. 1g).

### GB265/266/266T MsAbs promote interactions between T cells and cancer cells

**Figure 2:**
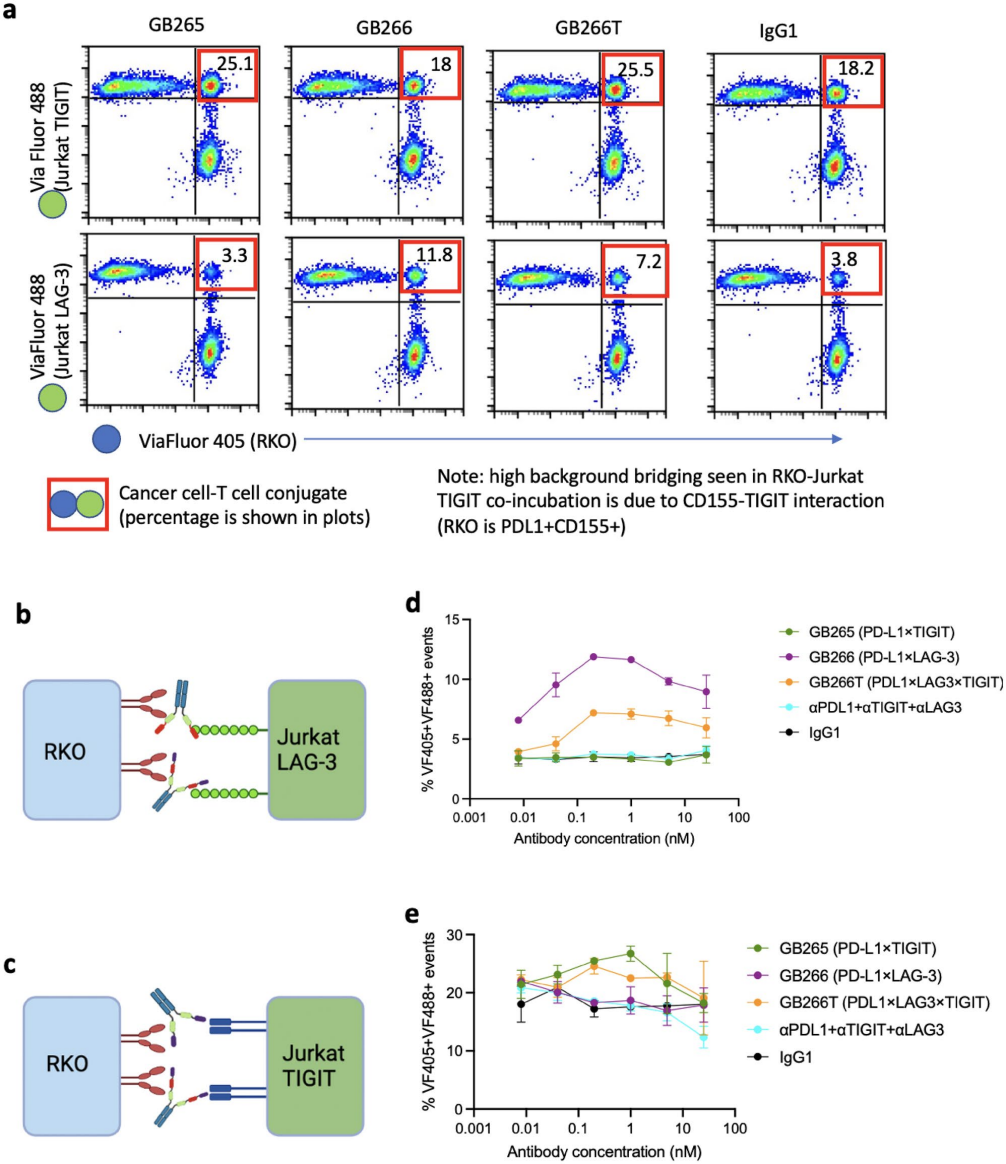
GB265/266/266T MsAbs mediate interactions between T cells and cancer cells. (**a**) Jurkat LAG-3 or TIGIT cells were labeled with ViaFluor 488, and incubated with RKO cells (PD-L1^+^CD155^+^) labeled with ViaFluor 405, in the presence of indicated antibodies (0.2 nM). Cell conjugates (events double-positive for the two fluorescent dyes. Boxed in red) were measured by flow cytometry. (**b**, **c**) Diagram showing the mechanism of GB265/266/266T-mediated tumor cell/T cell bridging. (**d**, **e**), Dose-dependent bridging of cancer cells with T cells by GB265/266/266T. Assays were performed in duplicates, and data are presented as mean ± SD.

One potential MOA of multispecific antibodies is to promote cell–cell interactions by bridging antigens expressed on respective cells. To test this possibility, we incubated cancer cells (RKO that express endogenous PD-L1) with Jurkat T cells that express LAG-3 (Fig. 2a, b) or TIGIT (Fig. 2c, d), in the presence of GB265, GB266, GB266T, or control monospecific antibodies. The cancer cells and the Jurkat cells were pre-labeled with different fluorescent dyes, and cell conjugates were identified as events that are positive for both fluorescent dyes using flow cytometry (Fig. 2a–c). Just as there is an optimal antibody/antigen ratio for the formation of polyclonal antibody-antigen complexes, we observed dose-dependent effects of the three multispecific antibodies on cancer cell-T cell conjugation, with maximal bridging between 0.2 and 1 nM, as compared to a combination of monospecific antibodies (Fig. 2d, e). At low concentrations, we observed that cell–cell bridging increased with antibody concentration until an optimal concentration is reached. Further increases in antibody concentration beyond this point lead to decreased cell conjugation, due to intense competition for target binding between crosslinked antibodies and excess free antibody molecules. These results demonstrate the dose-dependent ability of our three multispecific antibodies to potentiate interactions of cancer cells and T cells that express different target molecules.

### GB265/266/266T multispecific antibodies with wildtype IgG1 Fc promoted robust antitumor T cell response in vitro

GB265, GB266, and GB266T were designed to target major immune checkpoint molecules that are expressed by cancer cells, antigen-presenting cells (APCs), and T cells in anti-cancer immune responses. In vivo, an antitumor T cell response begins with the activation of T cells by tumor antigens that are presented to the T cell receptor (TCR) by APCs as peptide-MHC complexes^40^. In the TME, activated T cells kill tumor cells but eventually become dysfunctional (exhausted) under the persistent stimulation of tumor antigens^41,42^. To develop an in vitro assay that best predicts in vivo efficacy of the therapeutics antibodies, we mimicked multiple cell type interactions and T cell exhaustion in the TME that are amenable to antibody treatment. TCR-mediated immune synapse formation between the APC/target cell and T cell is required for efficient T cell activation, immune checkpoint function, and T cell cytotoxicity toward the target cell. We therefore first established a T cell-engaging cancer cell line by transfecting the human colon cancer cell line RKO, which expresses endogenous PD-L1 (a PD-1 ligand) and CD155 (a TIGIT ligand) but not MHC-II (a LAG-3 ligand), with a transmembrane form of anti-human CD3 antibody (OKT3) scFv to engage and activate the TCR. Tumor cells engineered with plasma membrane-expressed anti-CD3 mAb fragments have been used in similar assays^32,43^.

**Figure 3:**
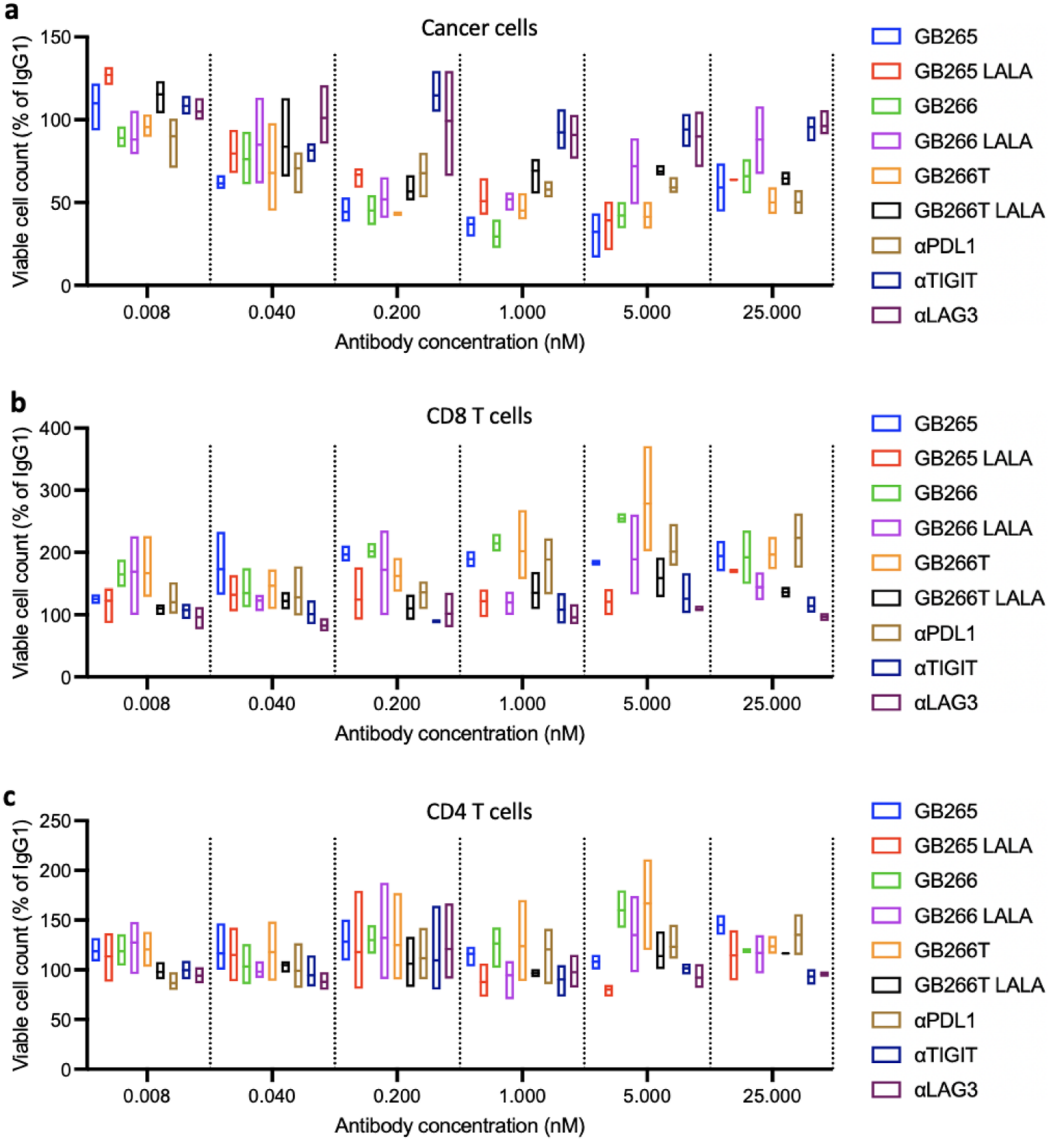
Dose-dependent promotion of cancer cell killing and T cell expansion by GB265, 266, and 266T in comparison with their Fc-deficient counterparts or parental antibodies. RKO OKT3 cells were co-cultured with human PBMCs, in the presence of serial dilutions of indicated antibodies. LALA denotes L234A/L235A mutations that disable Fc effector function. αPDL1, αLAG3, and αTIGIT are parental monospecific antibodies used for the construction of GB265, GB266, and GB266T. The number of viable cancer cells (**a**), CD8 T cells (**b**), and CD4 T cells (**c**) was determined by flow cytometry. Gating strategy is shown in Fig. S1. Assays were performed in triplicate and data are presented as box plots (min to max, with line at mean). Statistical significance was assessed using two-way ANOVA with multiple comparisons and results are shown in Table S1.

In co-culture of these cells (RKO OKT3) with human PBMCs, we found that GB265, GB266, and GB266T with wildtype IgG1 Fc outperformed parental antibodies in the promotion of cancer cell killing (Fig. 3a), CD8 T cell expansion (Fig. 3b), and CD4 T cell expansion (Fig. 3c). Fc binding to Fcγ receptors expressed on cells, such as monocyte/macrophages, NK cells, dendritic cells, and B cells, can have a profound impact on the efficacy of immune checkpoint-blocking antibodies^30^. Although Fc-silencing is a common practice in the development of therapeutic antibodies targeting checkpoint molecules on T cells to prevent T cell depletion by ADCC/ADCP/CDC, we found that LALA (L234A/L235A) mutants of the three multispecific antibodies had greatly reduced potency (Fig. 3a–c and Table S1), suggesting the importance of retaining Fc function for these antibodies. The results suggest that for therapeutic antibodies that engage T cells with tumor cells, intact Fc function combined with imbalanced binding affinities (relatively low T cell antigen binding and high tumor antigen binding) can effectively circumvent the issue of T cell depletion while potently promoting T cell activation and tumor cell killing.

**Figure 4:**
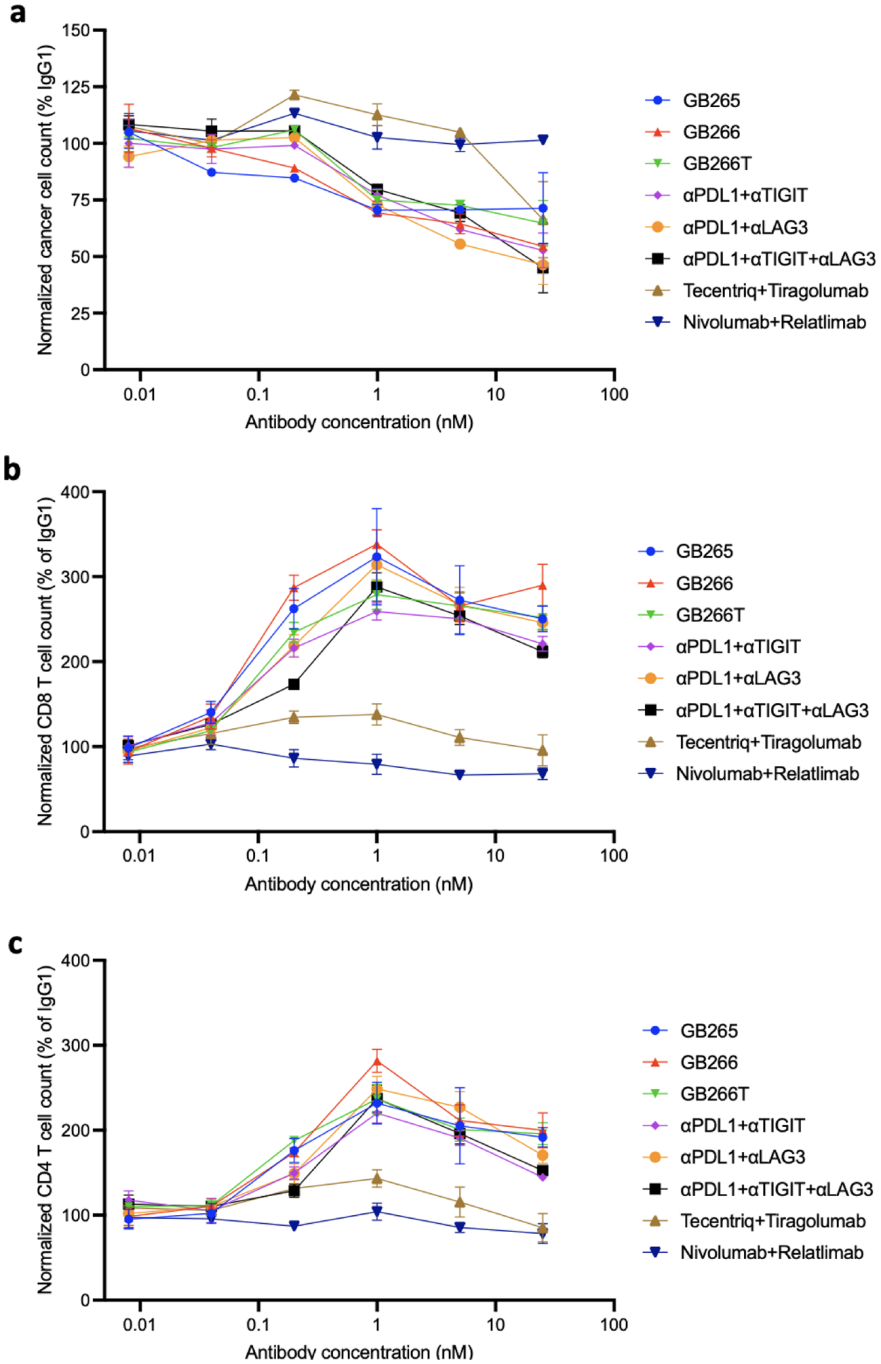
Dose-dependent promotion of cancer cell killing and T cell expansion by GB265, 266, and 266T. Raji PD-L1 CD155 OKT3 cells were co-cultured with human PBMCs, in the presence of serial dilutions of indicated antibodies. αPDL1, αLAG3, and αTIGIT are parental monospecific antibodies used for the construction of GB265 (Fc^+^ and Fc^–^), GB266, and GB266T. Number of viable cancer cells (**a**), CD8 T cells (**b**), and CD4 T cells (**c**) was determined by flow cytometry. Gating strategy is shown in Fig. S1. Assays were performed in triplicate and data are presented as mean ± SEM. Statistical significance was assessed using two-way ANOVA with multiple comparisons and results are shown in Table S2.

To test whether these antibodies are also efficacious with other cancer cells, we transfected the B cell lymphoma cell line Raji (FcγRIIB^+^PD-L1^−^MHC-II^+^CD155^−^) with OKT3, PD-L1, and CD155 and co-cultured the resultant cells (Raji OKT3 PDL1 CD155) with PBMCs in the presence of serial dilutions of test and control antibodies. We found that at antibody concentrations lower than 1 nM, our antibodies outperformed benchmark antibodies and parental antibody combinations in promotion of both cancer cell killing (Fig. 4a) and T cell expansion (Fig. 4b, c), correlating with cancer cell-T cell bridging (Fig. 2). At doses higher than 1 nM, they performed comparably with combinations of parental antibodies but better than benchmark antibodies, including nivolumab/relatlimab combination that has shown higher clinical activity than nivolumab monotherapy in untreated advanced melanoma^23^. These data suggest in addition to intact Fc function, GB265/GB266/GB266T-induced tumor cell-T cell bridging through PD-L1 on cancer cells and TIGIT/LAG-3 on T cells is a critical attribute for these antibodies to elicit efficient anti-cancer T cell response.

**Figure 5:**
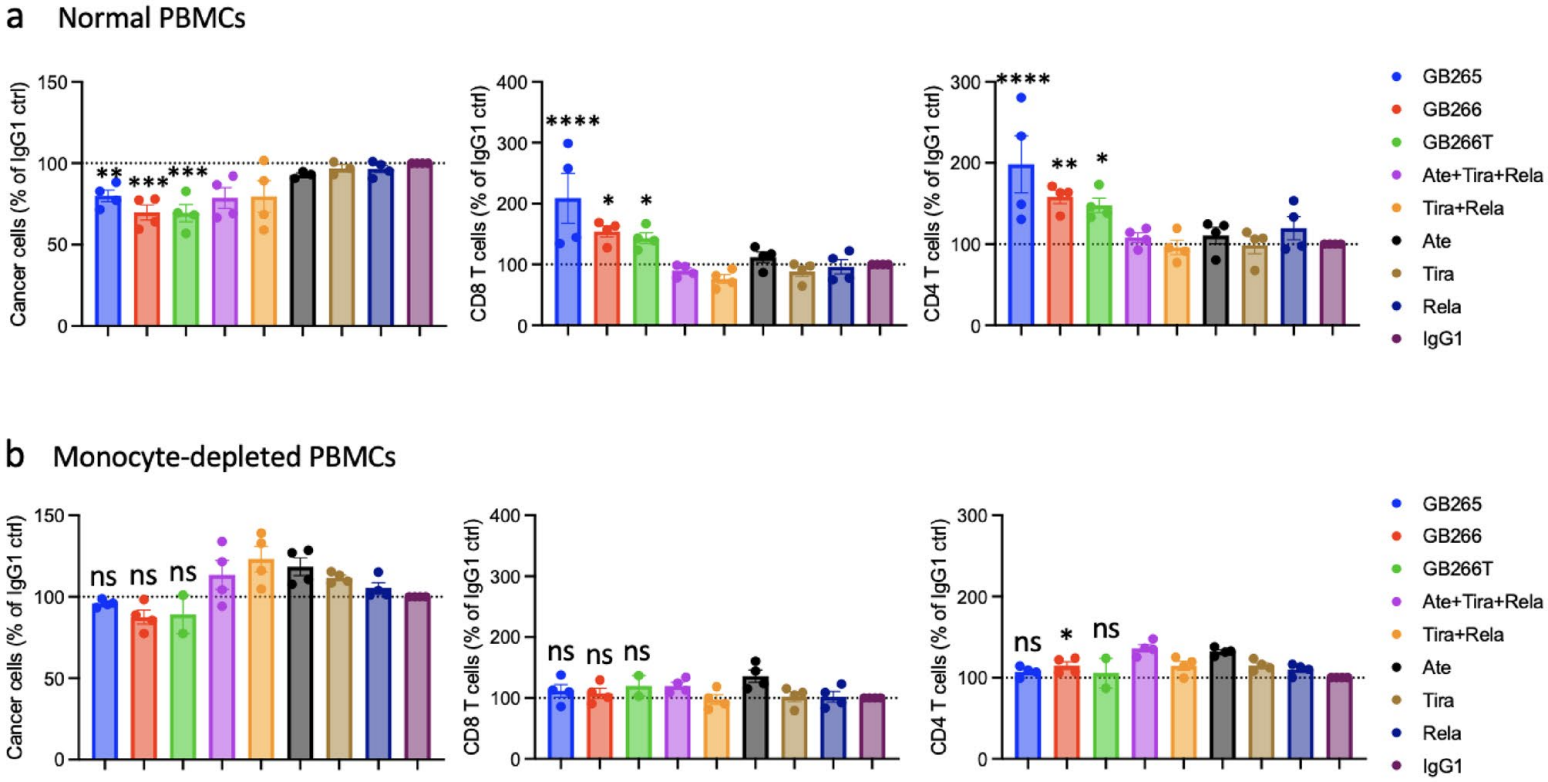
GB265, GB266, and GB266T antibody efficacy is monocyte-dependent. Raji PD-L1 OKT3 cells were co-cultured with normal (**a**) or monocyte-depleted (**b**) PBMCs in the presence of indicated antibodies at 30 nM for 4 days. Viable cell count of CD8 T cells, CD4 T cells, and cancer cells were determined by flow cytometry. Statistical significance of test antibodies versus IgG1 control was assessed using one-way ANOVA with Fisher’s LSD test for multiple comparisons and is shown by asterisks. *P < 0.05; **P < 0.01; ***P < 0.001; ****P < 0.0001. *ns* not significant.

Fc binding to Fcγ receptors expressed on cells, such as monocyte/macrophages, NK cells, dendritic cells, and B cells, can have a profound impact on the efficacy of immune checkpoint-blocking antibodies^30^. Although Fc-silencing is a common practice in the development of therapeutic antibodies targeting checkpoint molecules on T cells to prevent T cell depletion, Figs. 3c and 4 illustrate that wild-type Fc function in GB265/266/266T actually enhances both T cell expansion and anti-tumor efficacy. We next tried to identify immune cells that mediate such effects. In PBMCs monocytes is a major component that express FcγRs and could contribute to the superior performance of Fc^+^ GB265/266/266T antibodies. To confirm this, we compared antibody efficacy in co-cultures of cancer cells (Raji PD-L1 OKT3) with either normal PBMCs or monocyte-depleted PBMCs (Fig. 5; Fig. S2). We found that while GB265, GB266, and GB266T promoted greater T cell expansion than control IgG1 and monospecific antibody combinations in co-culture of T cells with normal PBMCs (Fig. 5a), their activities were largely mitigated when monocyte-depleted PBMCs were used in the co-culture (Fig. 5b). These results suggest the importance of monocytes for the efficacy of GB265, GB266, and GB266T. This is likely because these myeloid cells express multiple proteins that can mediate the function of these antibodies in T cell activation, such as FcγRs, PD-L1, CD155, MHC-II and co-stimulatory molecules that include CD80 and CD86.

Taken together, the in vitro data demonstrate that Fc-competent GB265, GB266, and GB266T are highly efficient in promoting T cell expansion and cancer cell killing when compared with benchmark antibody and antibody combinations through multiple MOAs including checkpoint blockade, Fc-mediated functions, and cancer cell-T cell bridging.

### GB265 and GB266T exhibit superior tumor suppression activity in animal models

**Figure 6:**
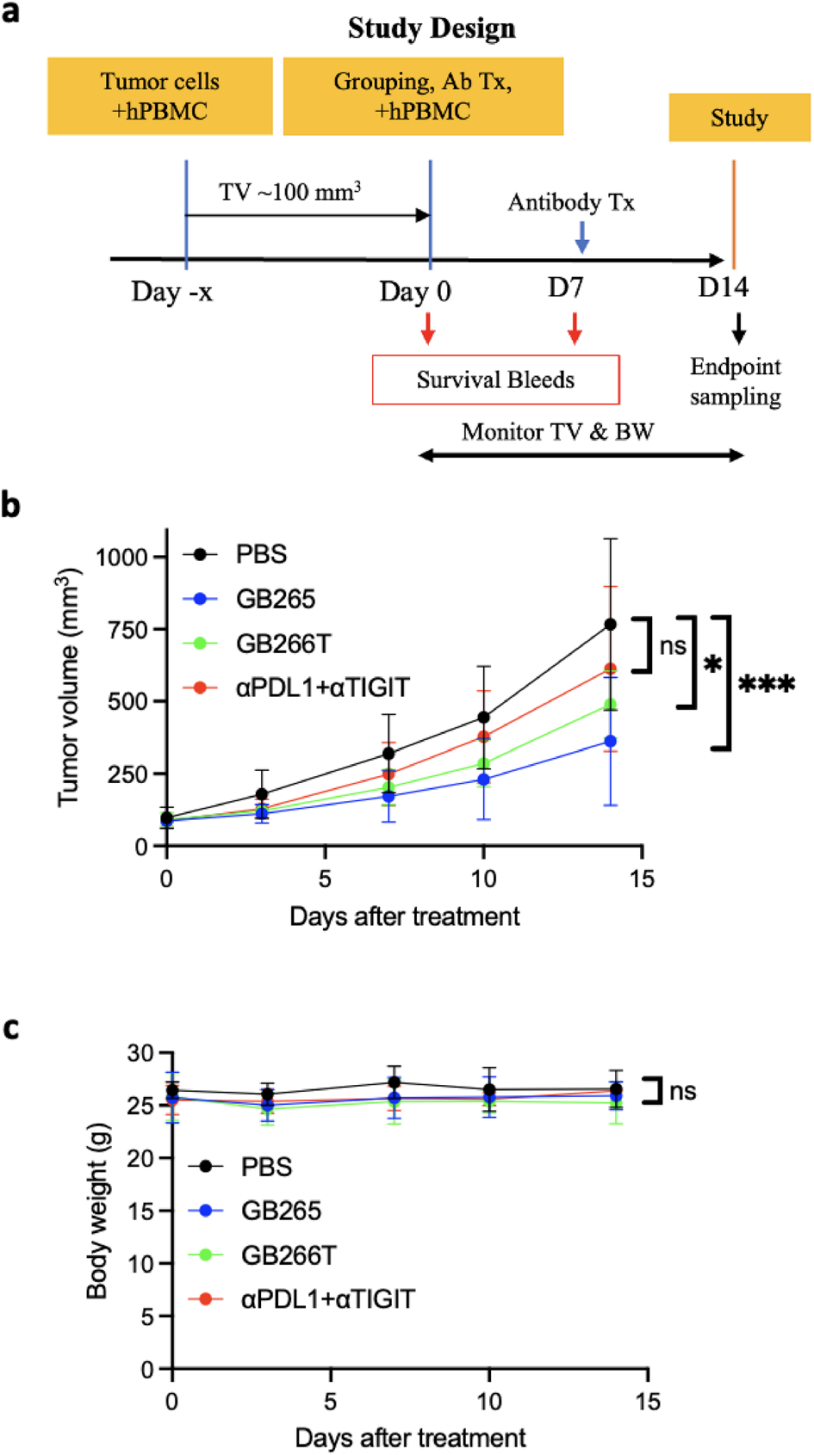
GB265 and GB266T efficiently suppress tumor growth in PBMC humanized animal model. (**a**) Schematic of study design. NSG mice were co-engrafted with RKO OKT3 cells and human PBMCs (i.v.). When tumors reached a volume of approximately 100 mm^3^ (day 0), mice were treated with PBS or indicated antibodies (5 mg/kg, i.v.) and another dose of PBMCs. Antibody treatment was repeated on day 7, and the experiment was terminated on day 14. Tumor volume (**b**), and animal body weight (**c**) were monitored during the treatment. Statistical significance on day 14 among treatment groups was assessed using two-way ANOVA with multiple comparisons (Tables S3 and S4). *P < 0.05; **P < 0.01; ***P < 0.001; ****P < 0.0001. *ns* not significant. Data represent mean ± SD of four mice in each group.

Encouraged by the results from in vitro cell-based assays, we further evaluated the antibodies in animal models. As these are humanized antibodies specific for human antigens, we used immunocompromised mice reconstituted with a human immune system and engrafted with human tumor cells. The reconstitution can be achieved by engraftment with human CD34^+^ hematopoietic stem cells or peripheral blood mononuclear cells (PBMC). We went with the PBMC reconstituted mouse model because it is the simplest and most cost-effective yet produces appropriate tumor-immune interactions, despite a shorter experimental window due to GvHD^44^. We engrafted PBMC-humanized NSG (NOD-scid IL2Rg^null^) mice with RKO OKT3 human colon cancer cells to promote T cell-tumor cell interactions (via CD3 engagement) and T cell exhaustion, as allogeneic T cells lack antigenic specificity for the engrafted tumor cells. We chose the PD-L1^+^ RKO cell line for this assay as all our test antibodies have the PD-L1-binding domain from envafolimab (Fig. 1a), and the RKO-engrafted humanized NSG mouse model has been successfully used to demonstrate the efficacy of anti-PD-L1 therapy^45^. The mice were subjected to antibody treatment after tumors were established, and tumor volume as well as body weight were monitored (Fig. 6a). We found that both GB265 and GB266T, but not the anti-PDL1/anti-TIGIT combination, elicited significant tumor growth suppression (Fig. 6b and Table S3), with no significant effects on body weight (Fig. 6c and Table S4). The results demonstrate the antitumor efficacy of GB265/GB266T in vivo with no appreciable safety concerns.

## Discussion

**Figure 7:**
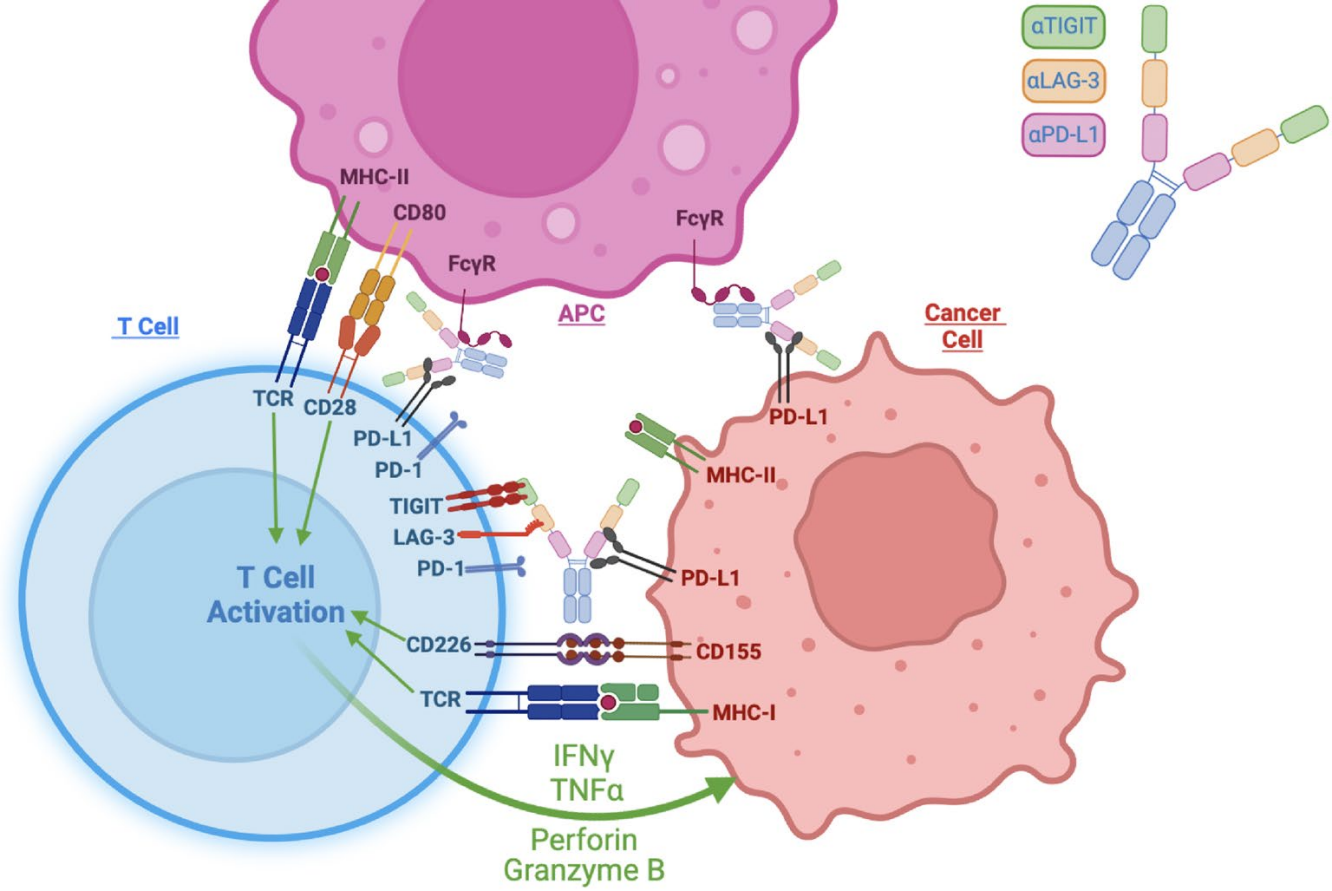
Schematic of the multiple mechanisms of action (MOA) for PDL1/LAG3/TIGIT multispecific antibodies. GB265, GB266, and GB266T antibodies engage T cells with cancer cells and APCs through Fc-FcgR interaction and antibody binding to target molecules PD-L1, LAG-3, and TIGIT, and block these major immune checkpoint pathways. All these MOAs work in concert to enhance T cell activation and tumor cell killing. Created with BioRender.com.

In this study we provide preclinical evidence that strongly supports the clinical development of multispecific checkpoint inhibitors targeting PD-L1, TIGIT, and LAG-3 as promising cancer immunotherapy agents. We show that an intact Fc is beneficial to their efficacy and that our three Fc-competent multispecific antibodies, GB265, GB266, and GB266T, outperform benchmark antibody combinations in anti-cancer T cell response in vitro and in an animal model of tumor suppression. Mechanistically, the data suggest that our antibodies promote T cell conjugation with tumor cells and APCs including monocytes through antibody-antigen binding and Fc-FcγR binding, and inhibit immune checkpoints, potentiating T cell activation and cancer cell killing (Fig. 7). Of particular interest, our data strongly argue that retaining intact Fc function for multispecific checkpoint antibodies is beneficial to cancer cell killing and T cell activation/expansion. These antibodies are also likely to have extended plasma half-lives in humans as binding of IgG Fc to neonatal FcR (FcRn) protects antibodies from catabolism^46,47^.

The engagement of Fc and FcγR can have a major effect on the efficacy of therapeutic antibodies through mechanisms that include antibody-directed effector activities and antibody-dependent signal augmentation via receptor clustering. Fc effector function can be a double-edged sword for its potential to deplete both undesired cells (e.g., cancer cells, Tregs) and desired ones (e.g., effector T cells), depending on the expression levels of the target proteins on different cells. Therefore, the decision to retain the Fc function of a therapeutic antibody needs to be carefully considered on a case-by-case basis. For anti-TIGIT antibodies in particular, whether it is beneficial to retain Fc binding for their therapeutic action has been a matter of debate^48^, but current results support the importance of Fc for the efficacy of anti-TIGIT antibodies in mouse models^22,32^. Our results from two PBMC co-culture systems with different cancer cells not only showed that intact Fc function of GB265, GB266, and GB266T did not lead to T cell depletion, they instead suggest that Fc function can be a critical contributor to the efficacy of these antibodies. This may be explained in part by their high affinity for PD-L1 on tumor cells but low to medium affinity for TIGIT and LAG-3 on T cells. These results will inform future development of similar multispecific checkpoint antibodies.

Our data further suggest that in co-culture assays of cancer cells with PBMCs the Fc effects of these antibodies on T cell activation and cancer cell killing are largely mediated by monocytes. Monocytes express MHC-II and T cell co-stimulatory molecules such as CD80 and CD86, and activation of FcγRI on these cells can further trigger their differentiation into immature DCs that induce T cell responses^49^. Monocytes and macrophages are myeloid cells that can be abundantly found in the TME of human cancers, and play important roles in the regulation of tumor progression and response to cancer immunotherapy^50^. It has been reported that myeloid cell FcγR engagement differentially affects anti-PD-1 and anti-PD-L1 antibody efficacy in vivo. While Fc-binding capacity compromises the antitumor activity of anti-PD-1 antibodies^51^, FcγR engagement augments the antitumor efficacy of anti-PD-L1 antibodies by altering myeloid subsets in the TME^31^. For anti-TIGIT antibodies, it was also observed that FcγR-mediated myeloid cell activation contributes to effective antitumor response^33^. We therefore anticipate that our Fc-competent anti-PDL1/LAG3/TIGIT multispecific antibodies GB265, GB266, and GB266T, which exhibited superior preclinical efficacy in an Fc- and monocyte-dependent manner, will be efficacious in the clinic as well. In human patients, the relative performance of the three antibodies is also expected to depend on cancer types and stages that present TMEs of diverse immune cell composition and immune checkpoint expression. While we observed no significant bodyweight changes in mouse tumor models treated with these antibodies, we will perform further toxicology evaluations, such as measurements of common immune-related adverse events (irAEs) in a species more closely related to humans (e.g., cynomolgus monkeys), before moving the programs forward to clinical stage.

Although there are other PD-L1 × TIGIT bsAbs under development, unlike GB265, none of them have been shown to outperform benchmark antibody combinations^52,53^. GB265 differentiates from most of those by retaining Fc function, which, as we show here, boosts antibody efficacy. While it was previously reported that Treg depletion is associated with anti-TIGIT efficacy^22^, ensuing studies found that the therapeutic effects of anti-TIGIT antibodies are independent of intratumoral Treg depletion^33^. Instead, a subset of CD8 T cells with high CD226 expression have been shown to be a prerequisite for anti-TIGIT immunotherapy^54^. As even intratumoral Tregs with high TIGIT expression are not depleted by treatment with Fc-competent anti-TIGIT antibodies^33^, concern about the depletion of effector T cells, which express markedly lower TIGIT than Tregs in the TME^22^, appears unfounded. Indeed, we did not see any evidence of effector T cell depletion by the Fc-competent GB265, GB266, or GB266T antibodies. Instead of T cell depletion, we found stronger T cell expansion and cancer cell killing after GB265/266/266T treatment, compared to controls. Lower TIGIT/LAG-3 binding affinities of these antibodies compared to their benchmarks could also prevent them from inducing ADCC/ADCP of effector T cells while maintaining their potency in promoting T cell activation and cancer cell killing.

GB266T is a first-in-class tsAb that targets PD-L1, TIGIT, and LAG-3, although Merck is working on a PD-1 × LAG-3 × TIGIT tsAb. We chose to target PD-L1 over PD-1 because by binding PD-L1 on cancer cells or APCs and TIGIT/LAG-3 on T cells, these antibodies can bridge APCs and cancer cells with T cells, improving the quality of immune synapses, and resulting in stronger T cell activation and tumor cell killing. Merck’s tsAb mainly targets inhibitory receptors on T cells and is expected to act only as a checkpoint inhibitor without the benefits of cell–cell bridging.

In summary, we have developed three multispecific antibodies (GB265, GB266, and GB266T) targeting PD-L1, TIGIT and/or LAG-3 that play the dual role of being both an immune checkpoint blocker and a T cell engager. Our work highlights the importance of intact Fc function and cancer cell-T cell bridging for the efficacy of these antibodies. Compared to combinations of monospecific checkpoint inhibitors, these single-molecule multispecific antibodies simplify clinical development and as our data indicated, are likely more efficacious in clinical settings as well. Roche’s Tecentriq/tiragolumab combination have recently failed a phase III trial, but BMS’s nivolumab/relatilimab combination has just been approved by the FDA for the treatment of unresectable or metastatic melanoma. Our data show that GB265, GB266, and GB266T have superior performance to these antibody combinations in assays that mimic the general characteristics of the TME, and could represent a better approach to target the PD-1, TIGIT and LAG-3 immune checkpoints in immunotherapy of cancers. Especially, our antibodies likely outperform current Fc-deficient checkpoint inhibitors in cancers such as B cell lymphoma and myeloid leukemia, where the cancer cells express Fcγ receptors, and Fc-competent multispecific checkpoint inhibitors can promote cancer cell-T cell interaction through both Ab-Ag binding and Fc-FcR binding.

## Methods

### Antibodies and cell lines

Therapeutic and detection antibodies used in this study are listed in Table S5 and S6, respectively. All cell lines were obtained from ATCC and maintained at 37 °C, 5% CO_2_, in RPMI-1640 supplemented with 10% fetal bovine serum (FBS) and 50 μM β-mercaptoethanol.

### Antibody construction, expression, and purification

All the antibodies are VHH type antibodies. Coding regions for the antibodies were synthesized and cloned into the expression vector pCDNA3.1 to generate the expression constructs, and their sequences were confirmed by sequencing.

For antibody expression and purification, the Expi293 cells were transiently transfected with above plasmids according to manufacturer’s instructions. Enhancers were added to cells 17 h after transfection. At 72 h after transfection, the cell culture was centrifuged at 3000*g* for 10 min. The supernatant was filtered with a 0.45 µm membrane and the antibody concentration was determined using Protein A probe on Gator (Probe Life). The VHH-Fc or the IgG antibodies were purified using Protein A columns on the AKTA Explorer 100 purification system (buffer A: PBS, pH = 7.4; buffer B: 0.1 M Glycine, pH = 2.5), and dialyzed in PBS twice. The antibodies were then filtered with a 0.22 µM membrane and used for experiments.

### Generation of stable cell lines

Transgenes were targeted to the AAVS1 safe harbor using the AAVS1 Transgene Knockin kit (Origene) with CRISPR-Cas9 knock-in technology for stable expression. Briefly, the coding region was amplified from template with primers (Table S7) by PCR using Q5 Hot Start High-Fidelity DNA Polymerase (NEB) and cloned into the donor vector pAAVS1-EF1a-Puro-DNR between the SalI and the MluI sites. The resultant donor construct was co-transfected with the pCas-Guide-AAVS1 vector (coding for Cas9 and AAVS1 guide RNA) into target cells by electroporation using the Neon system (Life Technologies). Puromycin selection for stable cell lines began 1 week after transfection and continued until untransfected control cells died off. Stable expression of the transgene was confirmed by flow cytometry with specific antibodies.

### Cell-based antibody binding assay

10^5^ cells (RKO, Jurkat TIGIT, and Jurkat LAG-3 for testing antibody binding to PD-L1, TIGIT, and LAG-3, respectively) per well were distributed in 0.1 ml medium to 96-well V-bottom plates. Supernatants were discarded after spinning down cells at 300*g*, 5 min at room temperature. Cells were resuspended in 50 μl of a serial dilution of antibodies and incubated for 1 h at room temperature. After incubation, cells were washed by adding 0.15 ml cell staining buffer (PBS containing 1% FBS and 0.1% NaN3) and centrifugation at 300*g*, 5 min at room temperature. Supernatants were discarded and cells were washed one more time with 0.15 ml of staining buffer. Cells were subsequently resuspended in 50 μl of secondary antibody (Alexa Fluor 647-conjugated AffiniPure Goat Anti-Human IgG, Fcγ Fragment Specific. Used at 1:500) and incubated at RT for 30 min. Cells were spun down after 0.15 ml cell staining buffer was added, resuspended in 0.12 ml of cell staining buffer, and analyzed by flow cytometry on a NovoCyte flow cytometer (Agilent).

### Cell-based antibody blocking assay

10^5^ cells (RKO for PD-1/PD-L1 blocking, Raji for MHC-II/LAG-3 blocking, and Jurkat TIGIT for CD155/TIGIT blocking) were distributed per well in 0.1 ml medium to 96-well V-bottom plate in duplicate. Supernatants were discarded after spinning down cells at 300*g*, 5 min at room temperature. Cells were resuspended in 50 μl of a serial dilution of antibodies containing 1 mg/ml of biotinylated probes (PD-1, LAG-3, or CD155) and incubated for 1 h at room temperature. After incubation, cells were washed by adding 0.15 ml cell staining buffer (PBS containing 1% FBS and 0.1% NaN3) and centrifugation at 300*g*, 5 min at room temperature. Supernatants were discarded and cells were washed one more time with 0.15 ml of staining buffer. Cells were subsequently resuspended in 50 μl of 1:500 Streptavidin-PE and incubated at RT for 30 min. Cells were spun down after 0.15 ml cell staining buffer was added, resuspended in 0.12 ml of cell staining buffer, and analyzed by flow cytometry on a NovoCyte flow cytometer (Agilent).

### Antibody-mediated cell–cell conjugation

Cancer cells (RKO cells that expresses endogenous PD-L1) and T cells (Jurkat cells transfeted with LAG-3 or TIGIT) were labeled with ViaFluor 405 (Bitotium) and ViaFluor 488 (Biotium), respectively, per the manufacturer’s instructions. Labeled cancer cells and T cells (5 × 10^4^ cells each per well) were combined, spun down, and resuspended in 50 μl of a serial dilution of antibodies and incubated for 1 h at room temperature. Cell staining buffer (0.1 ml) was added and samples were analyzed by flow cytometry to quantify VF405/488 double positive events that represent cancer cell-T cell conjugates.

### Monocyte depletion of PBMCs by adherence

Human PBMCs in 6-well tissue culture plate were incubated in 37 °C/5% CO_2_ incubator to allow monocyte adhesion. Unattached cells were harvested and monocyte frequency in PBMCs with or without depletion was determined by flow cytometry after staining with CD14 antibodies.

### Cell-based model of antitumor immune response for therapeutic antibody evaluation

Cancer cells transfected with a transmembrane form of anti-human CD3 antibody (OKT3) scFv were labeled with ViaFluor 405 (Biotium), as described above. Human PBMCs were washed in warmed medium (RMPI-1640 containing 10% FBS and 50 μM β-mercaptoethanol) and combined with labeled cancer cells (5 × 10^4^ PBMCs and 1 × 10^4^ cancer cells in 0.15 ml medium for each well). The cell mixture was then transferred to 96-well U-bottom plates containing 50 μl antibody and incubated in a 37 °C/5% CO_2_ incubator for 4 days. Cells were then harvested and stained with the Zombie NIR Fixable Viability Kit (BioLegend) per the manufacturer’s instructions. The cells were subsequently stained with FITC-labeled anti-human CD8 and APC-labeled anti-human CD4 antibodies (BioLegend) and analyzed by flow cytometry to quantify viable cancer cells (Zombie^–^VF405^+^), CD8 T cells (Zombie^–^CD8^+^) and CD4 T cells (Zombie^–^CD4^+^) (see Fig. S1 for gating strategy).

### Mouse tumor model and treatments

Animal work was outsourced to Biocytogen. All experimental protocols were approved by Biocytogen’s Institutional Care and Use Committee (IACUC). All methods were carried out in accordance with relevant guidelines and regulations set forth by The Association for Assessment and Accreditation of Laboratory Animal Care (AAALAC) International and are reported in accordance with ARRIVE guidelines.

NSG female mice at 6–8 weeks of age were subcutaneously injected with 5 × 10^6^ RKO OKT3 cells and 2.5 × 10^6^ human PBMCs in Matrigel (1:1) on the right flank. Two weeks later, when tumor size reached a volume of approximately 100 mm^3^ (day 0), mice were randomized into treatment groups and injected i.v. with 10^6^ human PBMCs and i.p. with 5 mg/kg body weight of GB265, GB266T, anti-PD-L1 (atezolizumab) plus anti-TIGIT (tiragolumab), or PBS, respectively. Body weight and tumor volume were measured twice weekly starting on day 0. Tumors were measured with a digital caliper and tumor size was calculated using the formula tumor volume (mm^3^) = L × W^2^/2.

### Statistical analysis

Unless noted otherwise, data visualization and statistical analyses were performed using Prism 9.4.0 (GraphPad). *P* values < 0.05 were considered significant. Statistical tests are specified in figure legends.

## Supplementary Information

Supplementary Information.

## Data Availability

Data and materials are available within the Article, Supplementary Information or from the corresponding author upon request.
