# Supplementary material for: Fc-competent multispecific PDL-1/TIGIT/LAG-3 antibodies potentiate superior anti-tumor T cell response
Source: Sci Rep. 2023 Jun 18;13:9865. doi: 10.1038/s41598-023-36942-3 (PMC10277278; doi:10.1038/s41598-023-36942-3)
Supplement: Supplementary file 1 — Supplementary Information. [file 41598_2023_36942_MOESM1_ESM.docx]

**Fc-competent multispecific PDL-1/TIGIT/LAG-3 antibodies potentiate superior anti-tumor T cell response**

Riyao Yang^1^, Su Huang^1^, Cai Huang^1^, Nathan S. Fay^2^, Yanan Wang^2^, Saroja Putrevu^2^, Kimberly Wright^2^, Mohd Saif Zaman^1^, Wenyan Cai^2^, Betty Huang^2^, Bo Wang^2^, Meredith Wright^2^, Matthew R. Hoag^2^, Allison Titong^2^, Yue Liu^1,2,^*

^1^Ab Therapeutics Inc., 3541 Investment Blvd., Suite 2, Hayward, CA 94545, USA.

^2^Ab Studio Inc., 3541 Investment Blvd., Suite 3, Hayward, CA 94545, USA.

*email: [yue.liu@antibodytherapeutics.com](mailto:yue.liu@antibodytherapeutics.com)


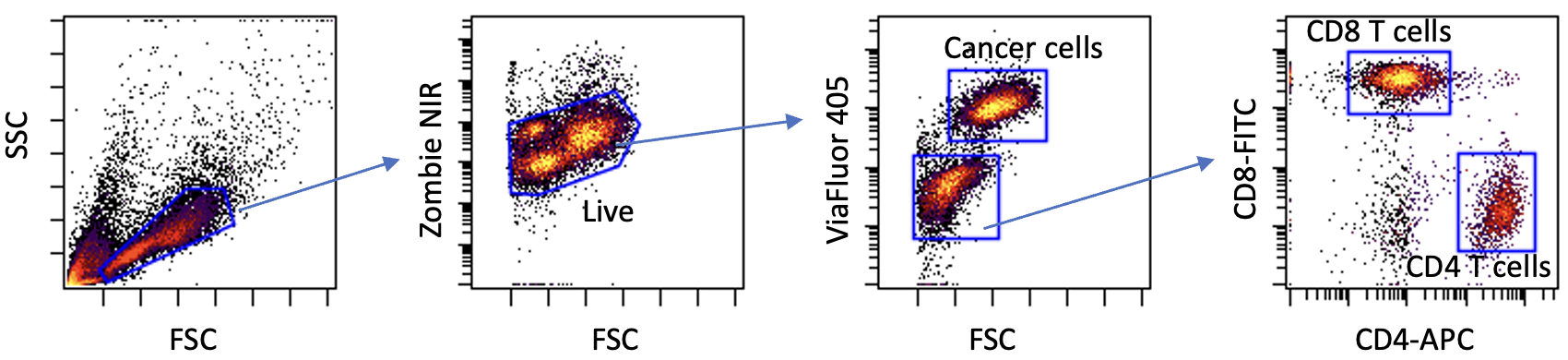


**Figure S1. Gating strategy for cancer cell/PBMC co-culture assays.**

**Table S1. Two-way ANOVA analysis of data in Fig. 3**


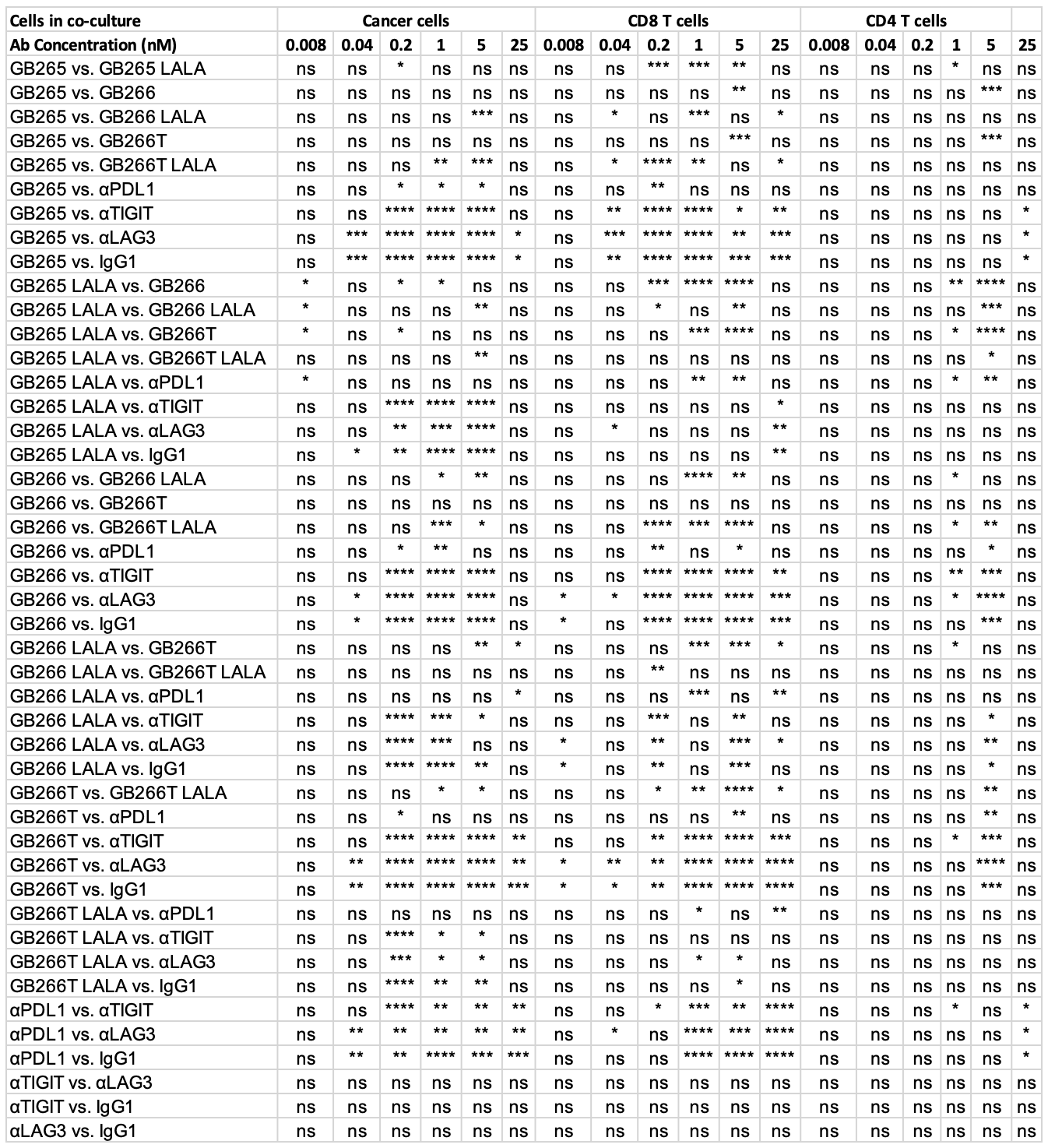


*P < 0.05; **P < 0.01; ***P < 0.001; ****P < 0.0001. ns, not significant.

**Table S2. Two-way ANOVA analysis of data in Fig. 4**


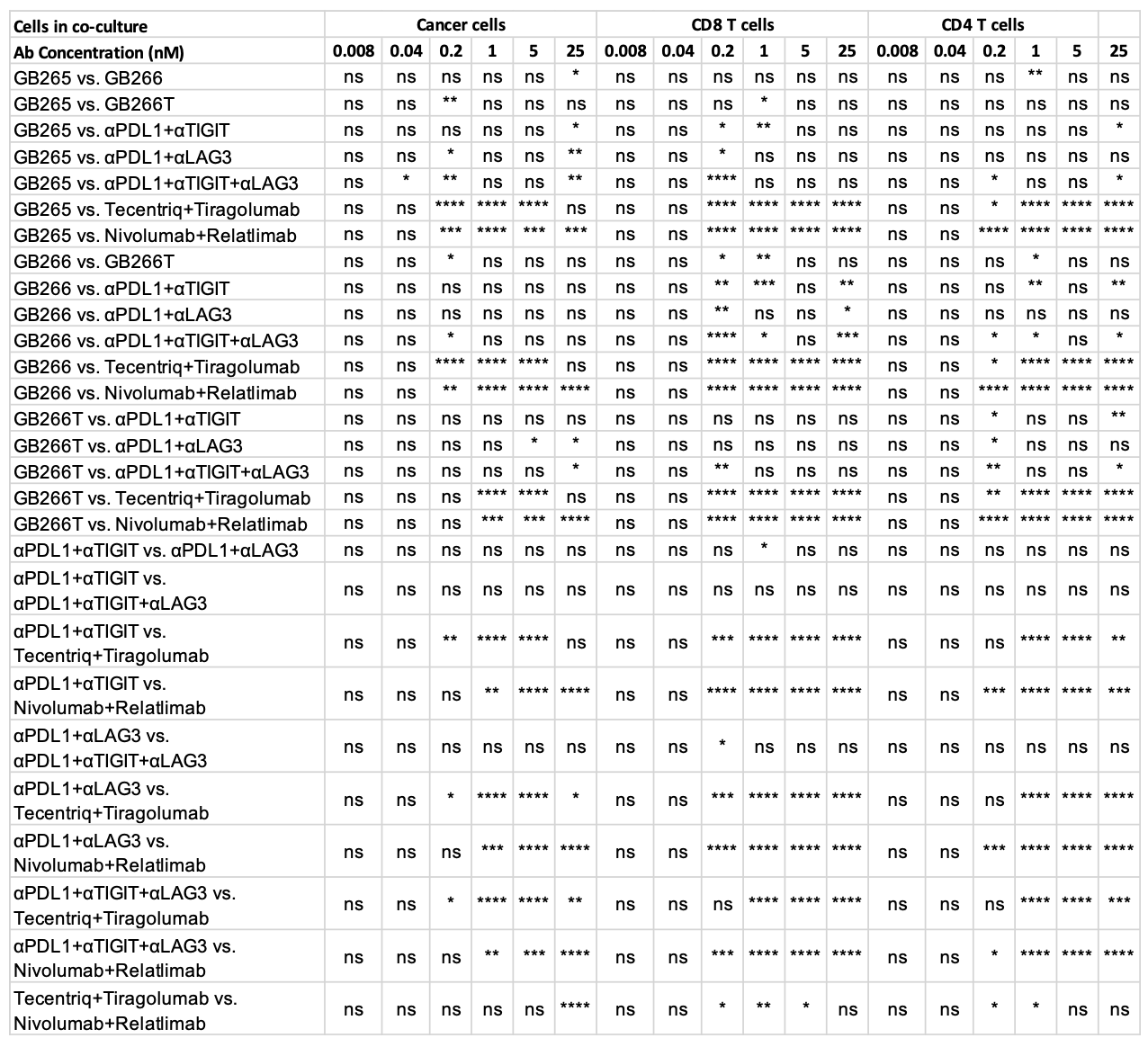


*P < 0.05; **P < 0.01; ***P < 0.001; ****P < 0.0001. ns, not significant.


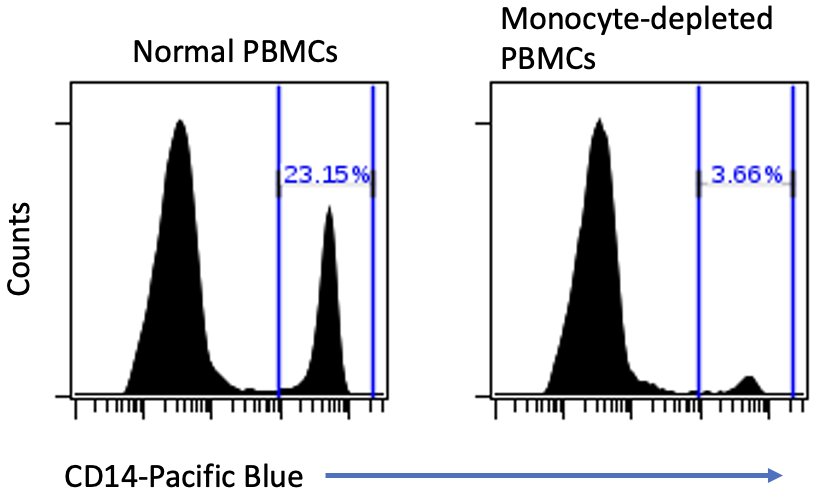


**Figure S2. Monocyte depletion of PBMCs by adherence.** Monocyte frequency in PBMCs with or without depletion was determined by flow cytometry after staining with CD14 antibodies.

**Table S3. Adjusted p-values from two-way ANOVA analysis of data in Fig. 6b**

**Table S4. Adjusted p-values from two-way ANOVA analysis of data in Fig. 6c**

**Table S5. IgG subclass and Fc status of therapeutic antibodies used in this study**

| **Antibody** | **Manufacturer** | **Target (s)** | **IgG subclass** | **Fc status** |
| --- | --- | --- | --- | --- |
| GB265 | ABT | PDL1xTIGIT | IgG1 | Intact |
| GB265 LALA | ABT | PDL1xTIGIT | IgG1 | Disabled |
| GB266 | ABT | PDL1xLAG3 | IgG1 | Intact |
| GB266 LALA | ABT | PDL1xLAG3 | IgG1 | Disabled |
| GB266T | ABT | PDL1xLAG3xTIGIT | IgG1 | Intact |
| GB266T LALA | ABT | PDL1xLAG3xTIGIT | IgG1 | Disabled |
| Envafolimab analog | ABT | PD-L1 | IgG1 | Intact |
| αLAG-3 | ABT | LAG-3 | IgG1 | Intact |
| αTIGIT | ABT | TIGIT | IgG1 | Intact |
| Atezolizumab | MCE | PD-L1 | IgG1 | Weakened |
| Relatlimab analog | ABT | LAG-3 | IgG1 | Intact |
| Tiragolumab | Selleck | TIGIT | IgG1 | Intact |
| Nivolumab | MCE | PD-1 | IgG4 | Intact |
| *InVivo*MAb human IgG1 isotype control | Bio X Cell | Unknown | IgG1 | Intact |

**Table S6. Other antibodies/reagents used in the study**

| REAGENT or RESOURCE | Supplier | Catalog # |
| --- | --- | --- |
| PE-Cy7 Anti Human CD155 | BioLegend | 337614 |
| Anti human CD4 APC | Invitrogen | 17-0049-42 |
| PE_Anti Human HLA-DR | BD-Pharmingen | 555561 |
| Alexa Fluor® 647 AffiniPure Goat Anti-Human IgG, Fcγ fragment specific | Jackson Immmuno Research Laboratories | 109-605-008 |
| Anti Human CD223(LAG-3) PE | Invitrogen | 12-2239-42 |
| Anti Human CD279 (PD-1) PE | Invitrogen | 12-2799-42 |
| Anti Human CD274 (PD-L1) APC | Invitrogen | 17-5983-42 |
| Streptavidin R-PE | Invitrogen | SA10041 |
| Anti Human TIGIT-PE | Invitrogen | 12-9500-42 |
| Pacific Blue™ anti-human CD279 (PD-1) | BioLegend | 329916 |
| Pacific Blue™ anti-human CD14 | BioLegend | 367122 |
| PE anti-human CD223 (LAG-3) | BioLegend | 369305 |
| FITC anti-human CD8a | BioLegend | 300906 |
| ImmunoCult Human CD3/CD28 T Cell Activator | StemCell Technologies | 10971 |
| Dynabeads™ Human T-Activator CD3/CD28 | ThermoFisher Scientific | 11131D |
| Human TruStain FcX™ (Fc Receptor Blocking Solution) | BioLegend | 422302 |

**Table S7. List of primers**

| Name | Sequence |
| --- | --- |
| CD155 FP | AAT TAT GTC GAC GCC ACC ATGGCCCGAGCCATGGCC |
| CD155 RP | AAT TAT ACG CGT CCT TGT GCC CTC TGT CTG TGG |
| PD-L1 FP | AAT TAT GTC GAC GCC ACC ATG CAG ATC CCA CAG GCG CC |
| PD-L1 RP | AAT TAT ACG CGT GAG GGG CCA AGA GCA GTG TC |
